# Supplementary material for: Lipid transporters E-Syt3 and ORP5 regulate epithelial ion transport by controlling phosphatidylserine enrichment at ER/PM junctions
Source: EMBO J. 2025 May 27;44(13):3697–719. doi: 10.1038/s44318-025-00470-9 (PMC12217353; doi:10.1038/s44318-025-00470-9)
Supplement: Supplementary file 1 — Appendix [file 44318_2025_470_MOESM1_ESM.pdf]

Appendix for

Lipid transporters E-Syt3 and ORP5 regulate epithelial ion transport by controlling phosphatidylserine enrichment at ER/PM junctions

Paramita Sarkar[1,4], Benjamin P. Lüscher[1,4,5], Zengyou Ye[1,4], Woo Young Chung[1], Ava Movahed Abtahi[1], Changyu Zheng[1], Min Goo Lee[2], Árpád Varga[3], Petra Pallagi[3], József Maléth[3], Malini Ahuja[1] and Shmuel Muallem[1,6]

From [1]Epithelial Signaling and Transport Section, National Institute of Dental Craniofacial Research, National Institutes of Health, Bethesda, MD 20892, USA, [2]Department of Pharmacology, Brain Korea 21 PLUS Project for Medical Sciences, Severance Biomedical Science Institute, Yonsei University College of Medicine, Seoul 03722, Korea, and [3]First Department of Internal Medicine, University of Szeged, Szeged, Hungary.

Table of contents:

Appendix Figures S1-S18 pages 2-19

Appendix Table S1, pages 20-24

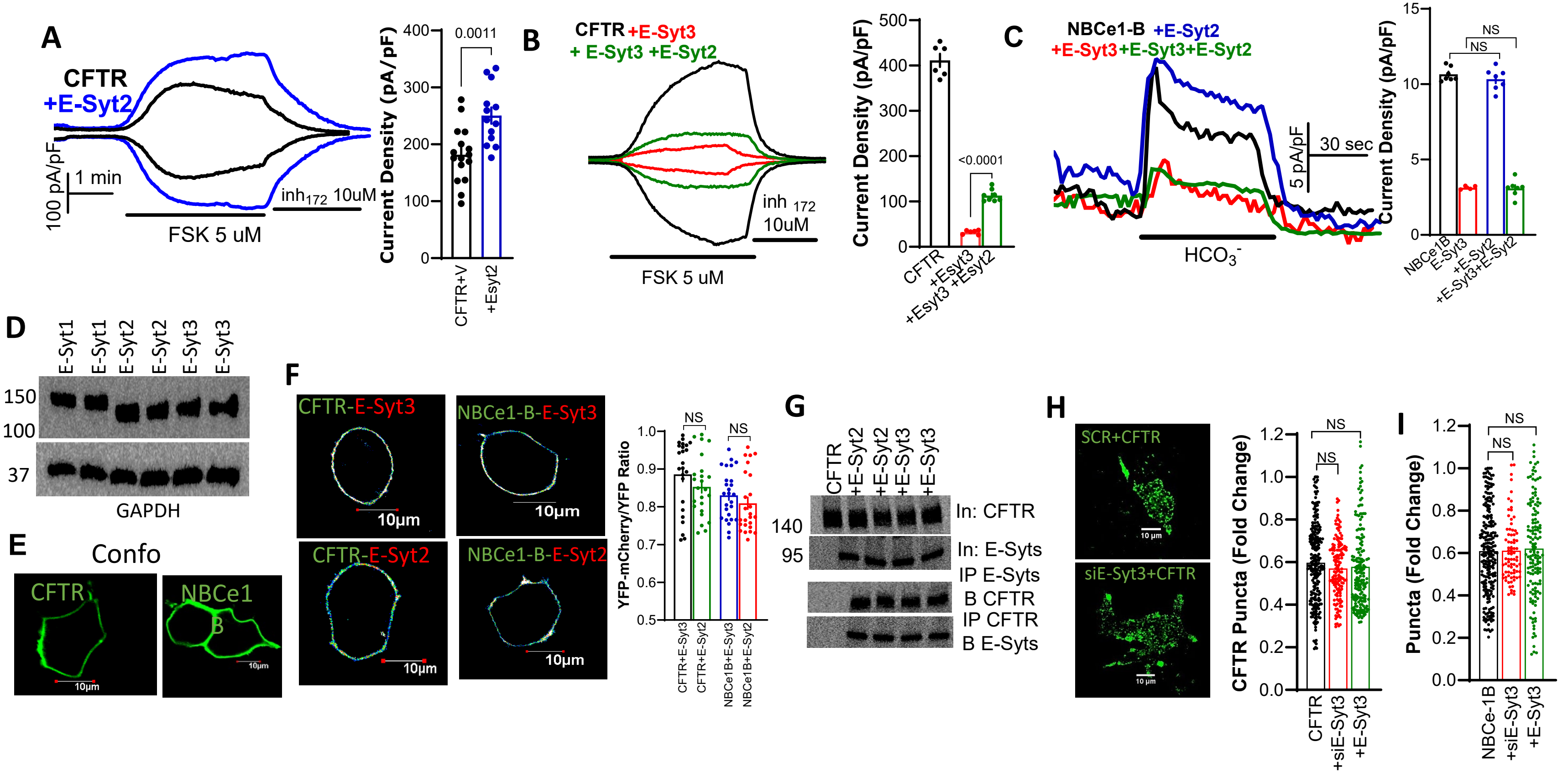

Appendix Figures S1: Effect of E-Syt2 on CFTR and NBCe1-B current, their interaction and localization.

(A-C) Current was measured in cells transfected with the same concentrations of CFTR (B, C), NBCe1-B (D) and E-Syt3 (C-D) as in Figure 1, by with 3 times higher concentration of E-Syt2 plasmid (B-D blue and Green).
(D) Expression level of the E-Syts and the corresponding GAPDH
(E) Example confocal images of cells expressing CFTR or NBCe1-B.
(E) Example images and summary of FRET between CFTR-GFP and NBCe1-B-GFP and either E-Syt3-mCherry or E-Syt2-mCherry
(G) Duplicate cells expressing CFTR, E-Syt2 and E-Syt3 were used for reciprocal Co-IP of CFTR and E-Syts.
(H-I) Effect of expression and depletion of E-Syt3 on localization of CFTR (H) and NBCe1-B (I) at the TIRF field.

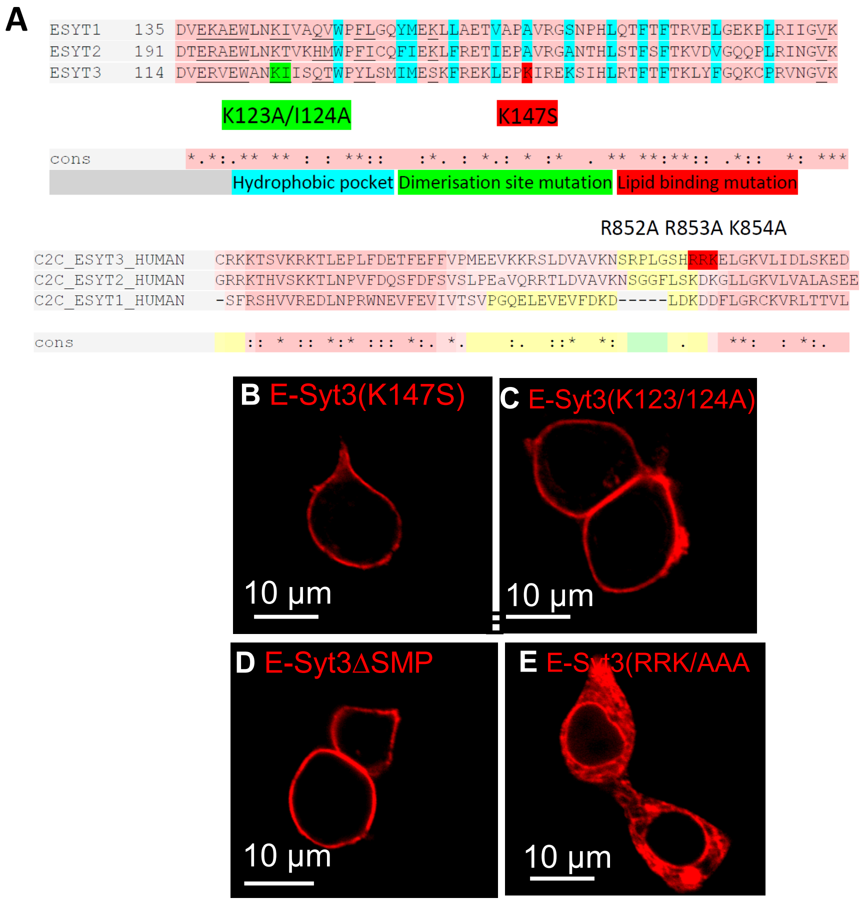

Appendix Figures S2: The E-Syt3 mutants used, localization of E-Syt3 mutants and role of the CFTR and NBCe1-B PDZ ligands on inhibition by E-Syt3.

(A) Alignment of the indicated regions of E-Syt1, E-Syt2 and E-Syt3 and the mutations used.
(B-E) Localization of the indicated E-Syt3 mutants used in the present studies.

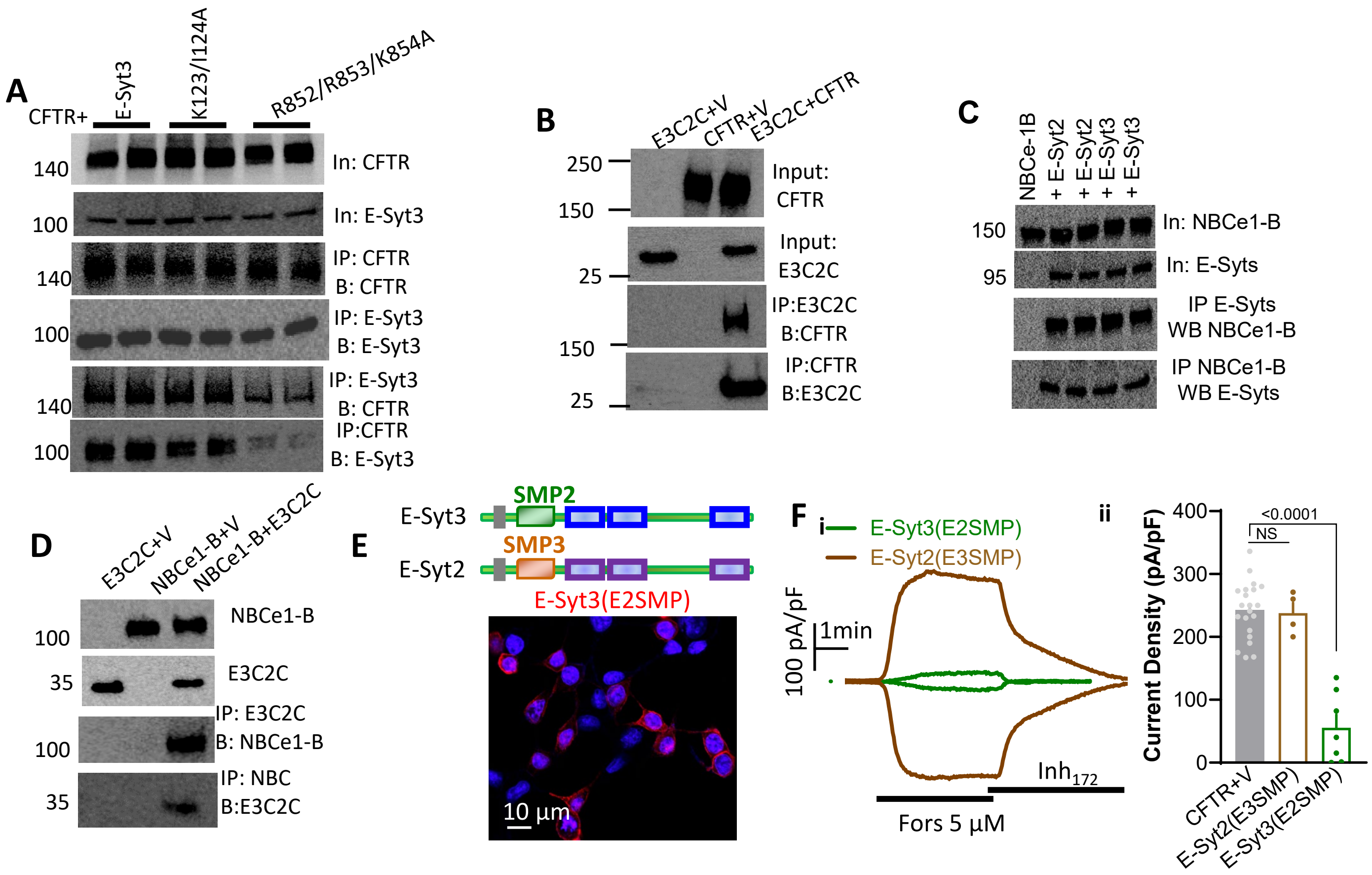

Appendix Figures S3: Co-IP of CFTR and NBCe1-B with the E-Syts and effect of E-Syt3 and E-Syt2 SMP switch of CFTR current.

(A) Duplicate cells expressing CFTR and E-Syt3, E-Syt3(K123/I124A) or E-Syt3(R852/R853/K854A) were used for reciprocal Co-IP of CFTR and E-Syt3.
(B) Cells expressing E3C2C+vector, CFTR +vector or CFTR+E3C2C were used for reciprocal Co-IP of CFTR and E3C2C.
(C) Duplicate cells expressing NBCe1-B, E-Syt2 and E-Syt3 were used for reciprocal Co-IP of NBCe1-B and E-Syts.
(D) Cells expressing E3C2C+vector, NBCe1-B+vector or NBCe1-B+E3C2C were used for reciprocal Co-IP of NBCe1-B and E3C2C.
(E) The E-Syt2 and E-Syt3 chimera with swapped SMP domain and localization of E-Syt3(E2SMP).
(F) Current of CFTR with E-Syt3(E2SMP) and E-Syt2(E3SMP) with the time course in (i) and current density in (ii).

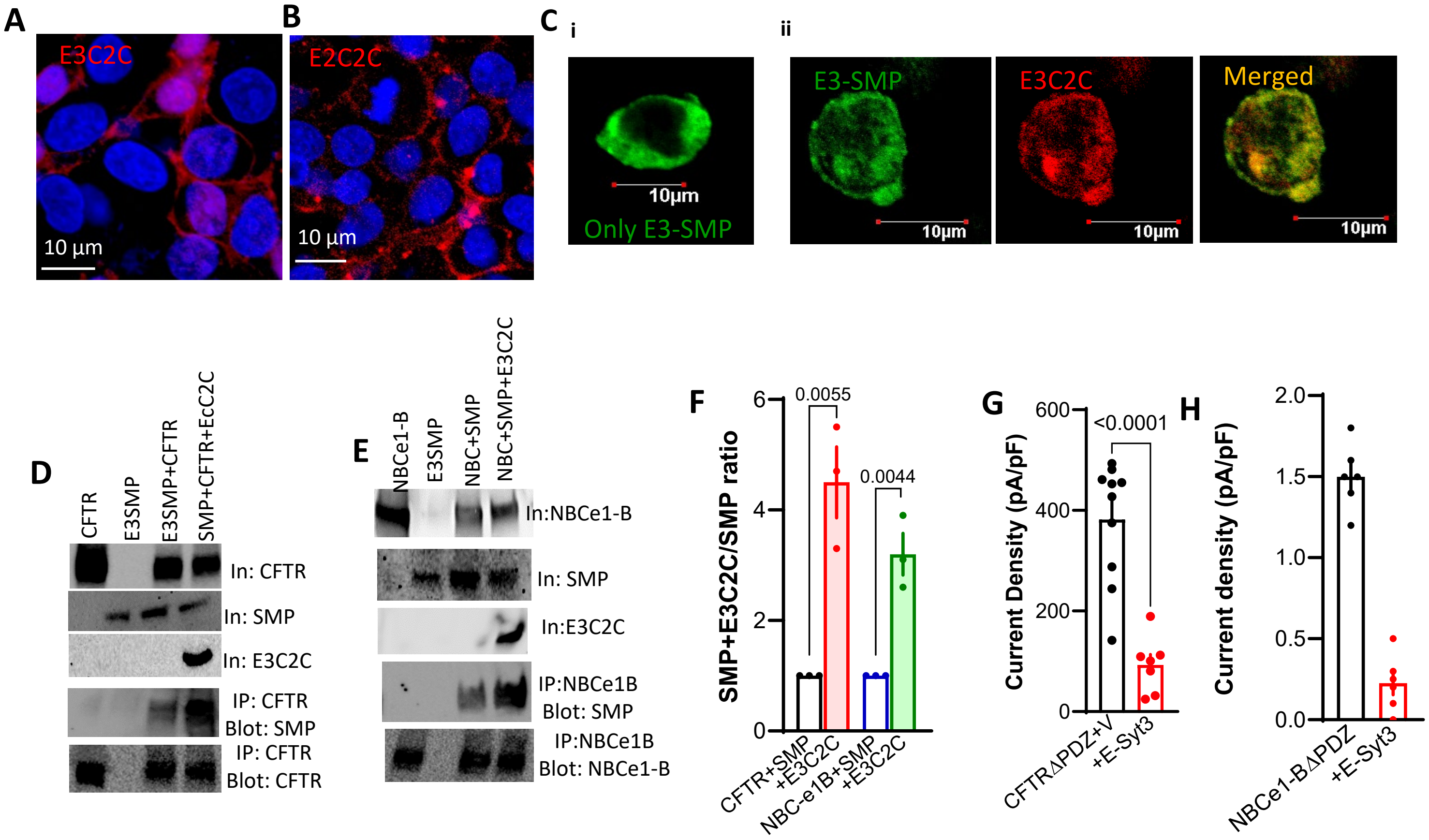

Appendix Figures S4: E3C2C targets the SMP domain to the CFTR and NBCe1-B at the PM.

(A-C) Localization of E3C2C (A) and E2C2C (B), E3SMP alone (Ci) and E3SMP together with E3C2C (Cii).
(D-F) Example of cells expressing CFTR, E3SMP and with and without E3C2C (D) or NBCe1-B, E3SMP and with or without E3C2C (E) were used to measure their Co-IP, as indicated. Summaries of 3-4 experiments are given in (F).
(G, H) Deletion of CFTR (G) and NBCe1-B (H) PDZ ligands had no effect on inhibition by E-Syt3.

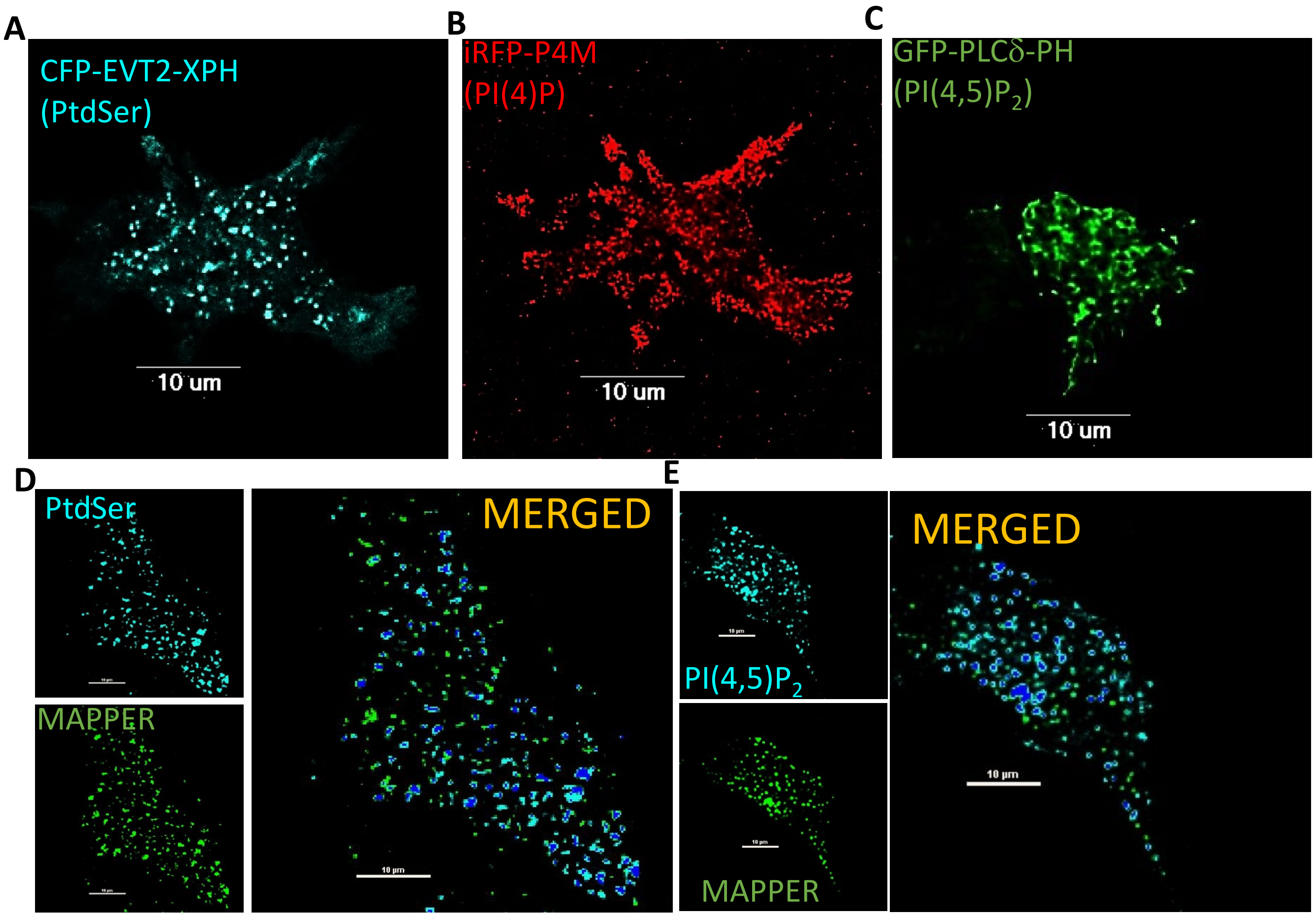

Appendix Figures S5: The probes used to measure PtdSer PI(4)P and PI(4,5)P_2 at the TIRF field.

(A-C) Example TIRF images of cells expressing the CFP-EVT2-XPH PtdSer sensor (A), iRFP-P4M PI(4)P sensor (B), and the GFP-PLCδ-PHD PI(4,5)P_2 sensor (C).

(D, E) Co-localization of the PtdSer and PI(4,5)P_2 sensors with the ER/PM junction marker MAPPER.

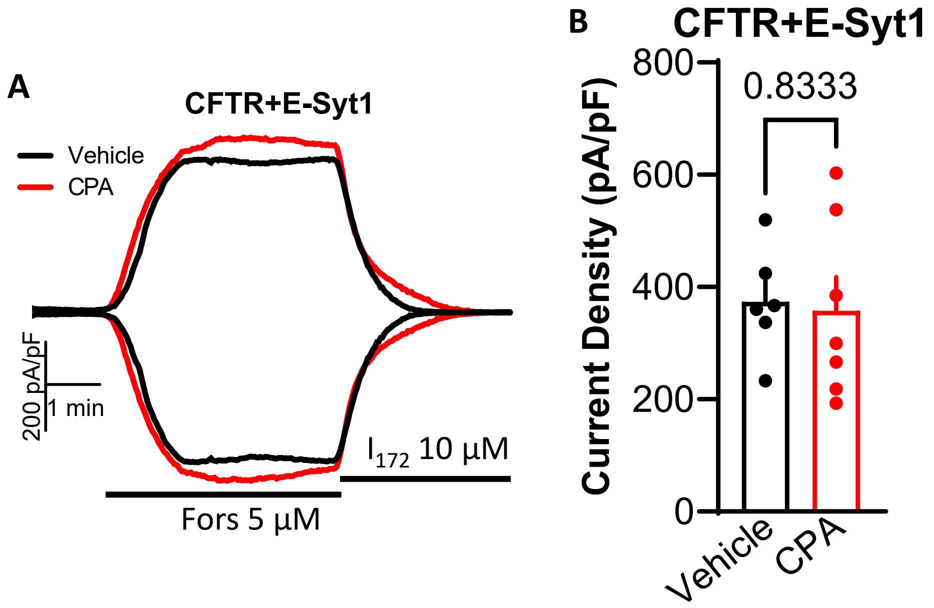

Appendix Figures S6: Effect of the activated E-Syt1 and massive PtdSer externalization on CFTR current.

Cells expressing CFTR+E-Syt1 were treated with vehicle (black) or 25 µM CPA for 15 min (red) to both activate E-Syt1 and cause massive PtdSer externalization (see Figure S4). No effect on CFTR activation and activity was noted.

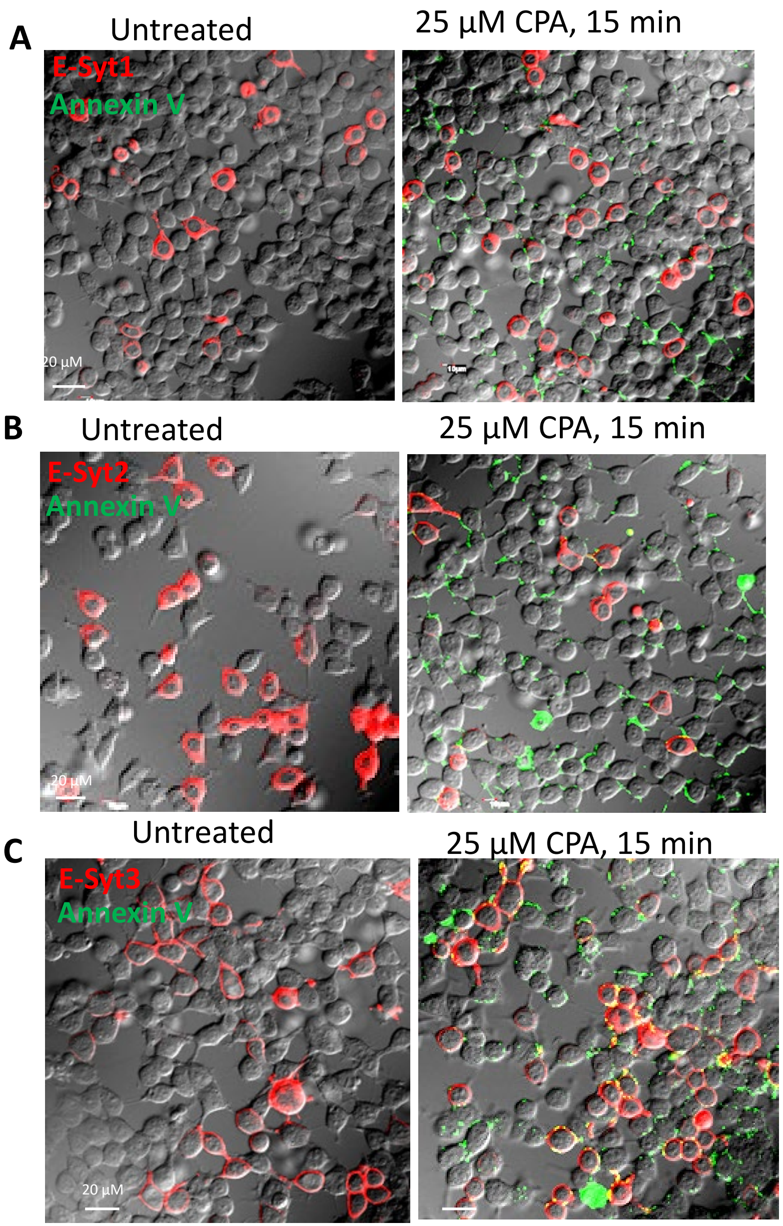

Appendix Figures S7 : Effect of the E-Syts on PtdSer externalization.

(A-C) Cells expressing E-Syt1 (A), E-Syt2 (B) or E-Syt3 (C) were untreated or treated with 25 µM CPA for15 min and stained with Annexin V to detect externalized PtdSer (green). The images represent results of 3 similar experiments.

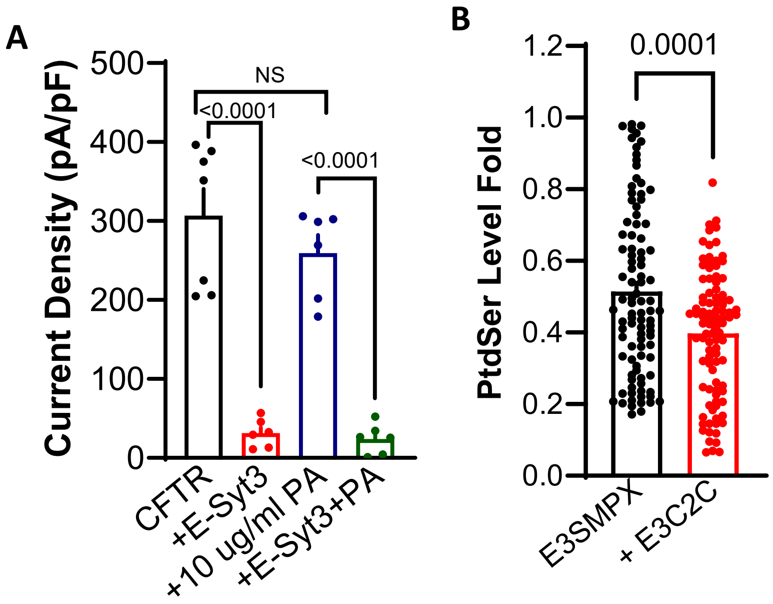

Appendix Figures S8: Effect of lipids on inhibition by E-Syts SMPs±E3C2C.

(A) CFTR current was measured in cells transfected with E-Syt3 (red) and infused with phosphatidic acid (PA, blue and green).

(B) Effect of E-Syt3X alone and when expressed with E3C2C on plasma membrane PtdSer.

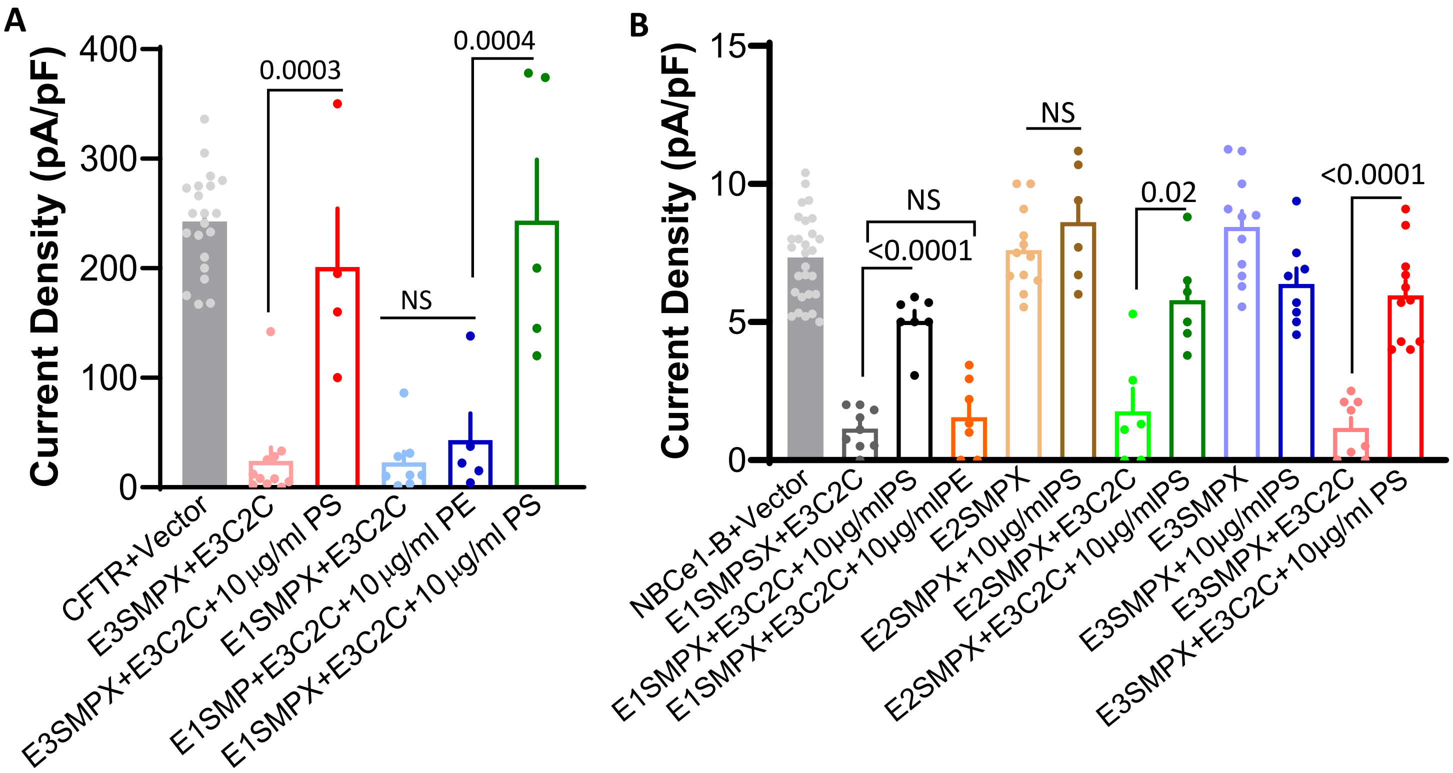

Appendix Figures S9: Effect of SMPX±E3C2C on CFTR and NBCe1-B current.

(A-B) Effects of infusing the cells with 10 µg/ml of PtdSer, PtdE or PtdC, as indicated, on inhibition of CFTR (A) or NBCe1-B (B) by E-Stys-SMPX alone of with E3C2C, as indicated in the columns. Note that only PtdSer reversed the inhibitions.

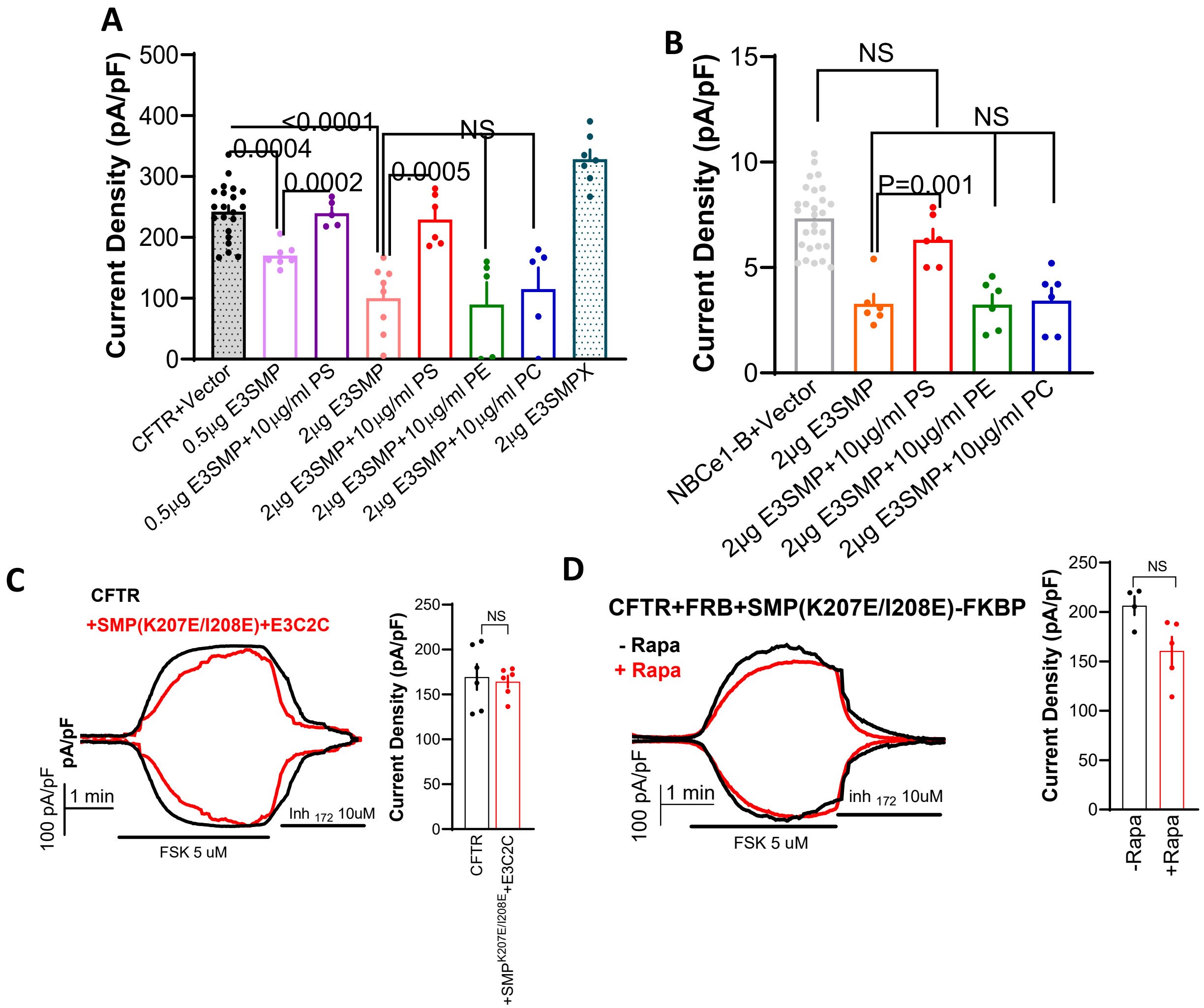

Appendix Figures S10 : Effect of SMPX±E3C2C and SMP(K207E/I208E) on CFTR and NBCe1-B current.

(A-B) Effects of infusing the cells with 10 µg/ml of PtdSer, PtdE or PtdC, as indicated, on inhibition of CFTR (A) or NBCe1-B (B) by E-Stys-SMP alone or with E3C2C, as indicated in the columns. Note that only PtdSer reversed the inhibitions.
(C-D) Effect of SMP(K207E/I208E) domain basic patches mutant on inhibition of CFTR by E3C2C (C) and by targeting to the FRB by 8 min pretreatment with rapamycin (D).

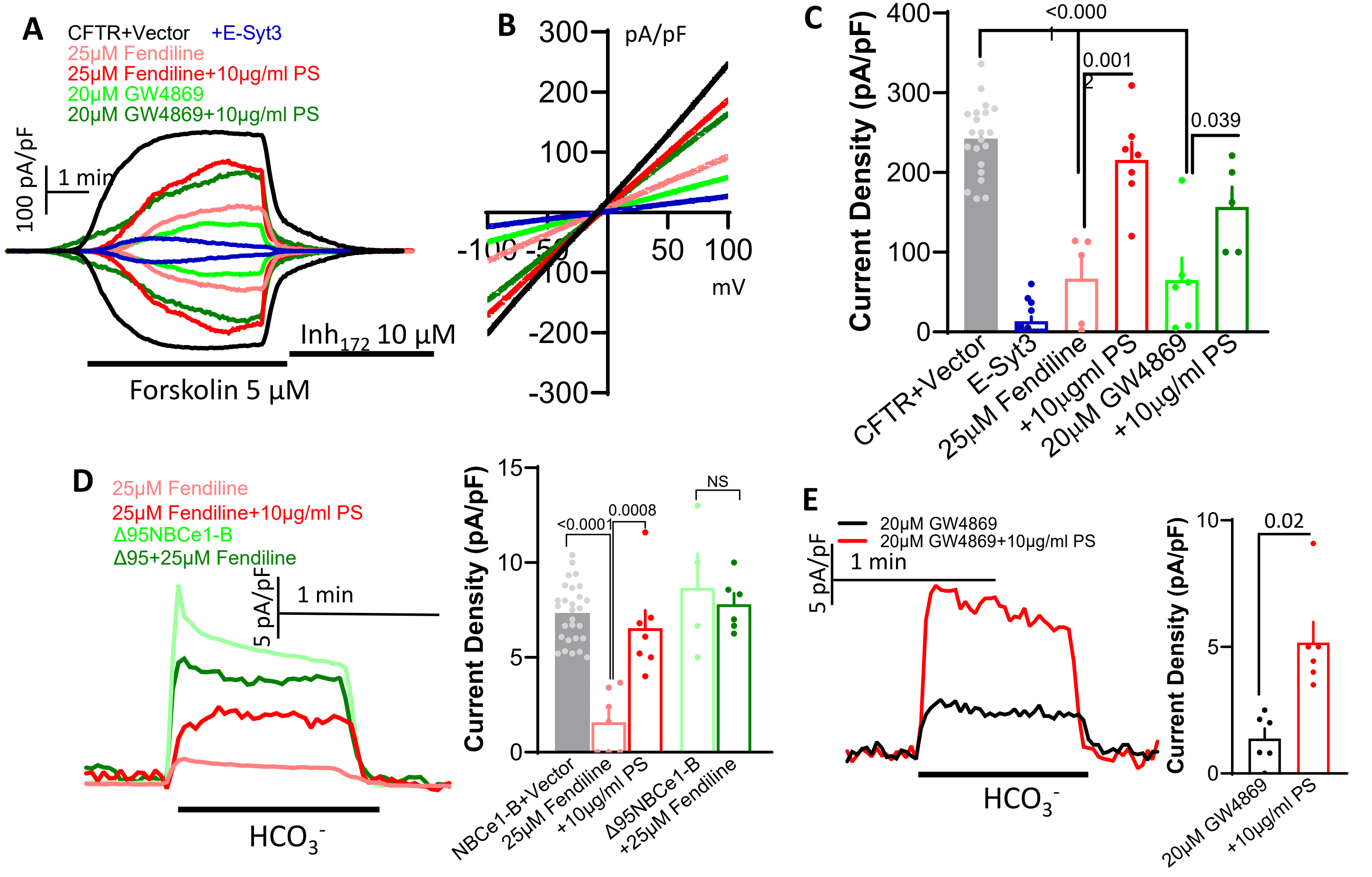

Appendix Figures S11: Effect of pharmacological reduction of plasma membrane PtdSer on CFTR and NBCe1-B current and reversal by PtdSer supplement.

(A-C) CFTR current was measured in cells transfected with CFTR and treated with 25 µM Fendiline (orange) or 20 µM GW4869 (green) for 30 min and then infused through patch pipette with vehicle (orange, green) or 10 µg/ml PtdSer (red, dark green) before measurement of CFTR current.
(D-E) NBCe1-B current was measured in cells transfected with NBCe1-B and treated with 25 µM Fendiline (D) or 20 µM GW4869 (F) for 30 min and then infused through patch pipette with vehicle or 10 µg/ml PtdSer, as indicated, before measurement of NBCe1-B current. Cells were also transfected with (Δ1-95)NBCe1-B and treated with Fendiline (D, E). (D, F) are example traces and (E, G) are the summaries.

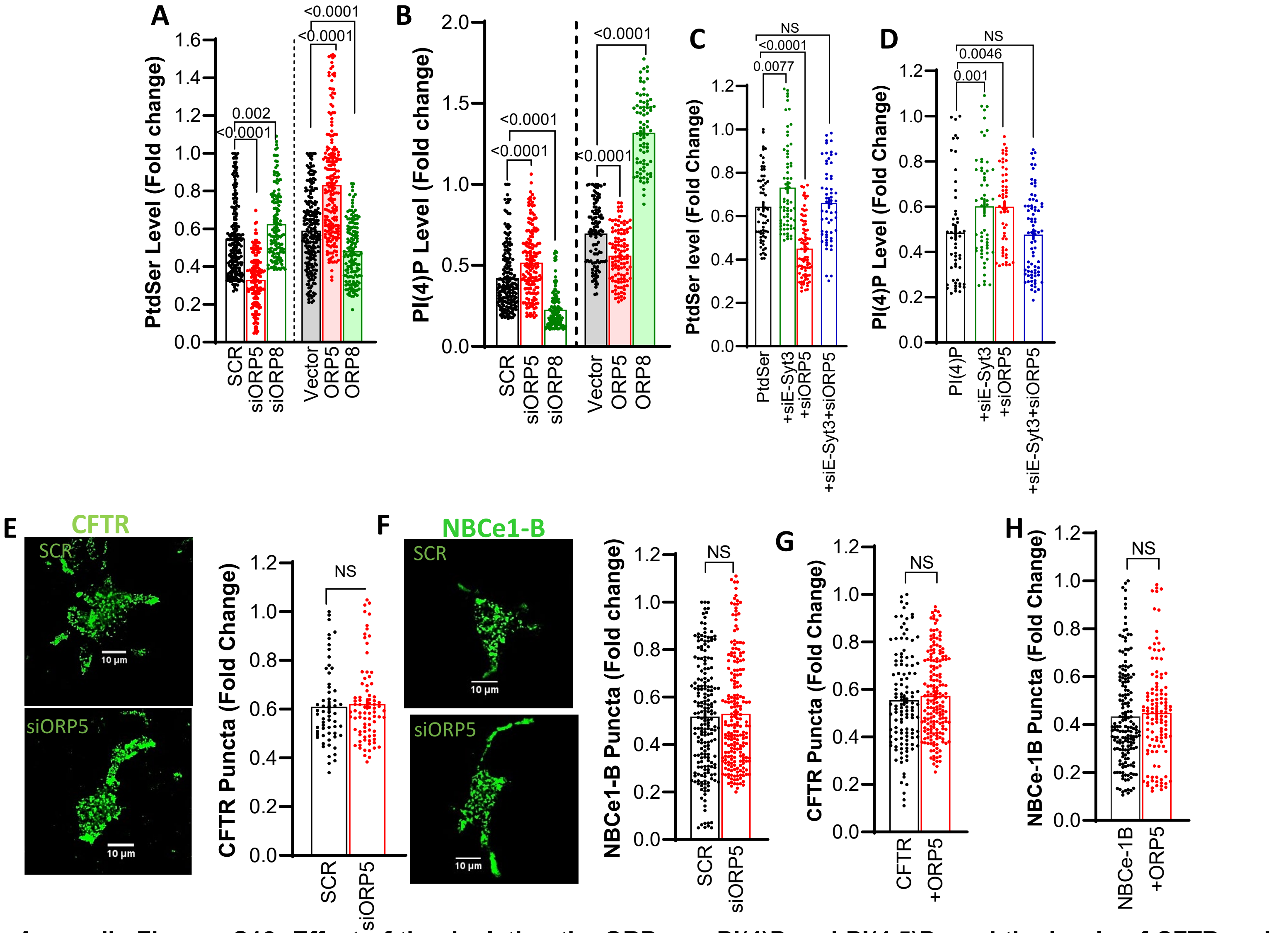

Appendix Figures S12: Effect of the depletion the ORPs on PI(4)P and PI(4,5)P_2 and the levels of CFTR and NBCe1-B at the TIRF field

(A, B) Effects of siORP5 and siORP8 (open columns), and ORP5 and ORP8 (filled columns) on junctional PtdSer (A) and PI(4)P (B).
(C, D) Effect of siORP5, siE-Syt3 and siORP5+siE-Syt3 on junctional PtdSer and PI(4)P.
(E, F) Effect of siORP5 on localization of CFTR (E) and NBCe1-B (F) at the TIRF field.
(G, H) Effect of ORP5 on localization of CFTR (G) and NBCe1-B (H) at the TIRF field.

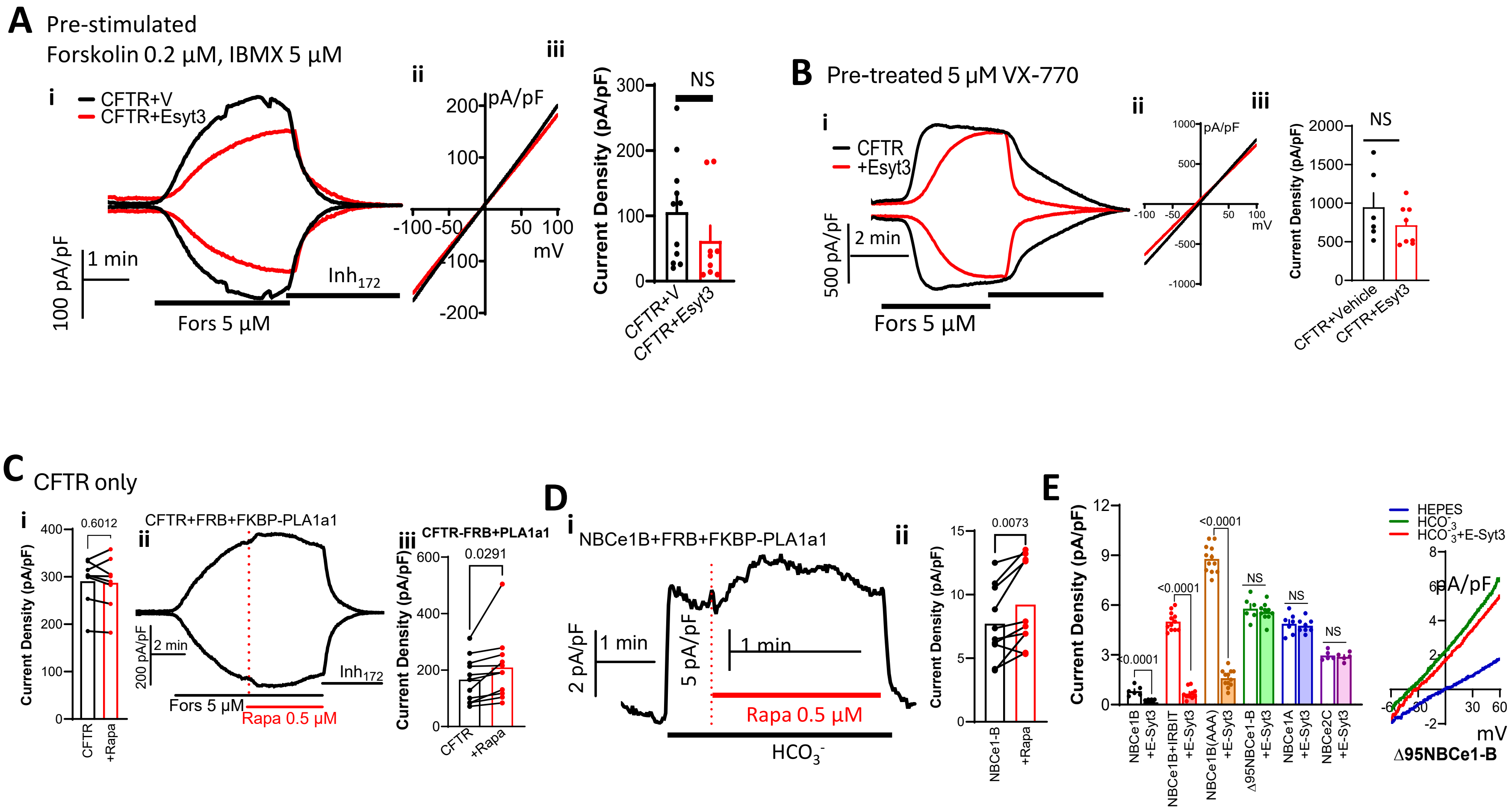

Appendix Figures S13: Effect of pre-stimulation of CFTR and late PtdSer depletion on inhibition by E-Syt3.

(A, B) Cells expressing vector (black) or E-Syt3 (red) and CFTR (A, B), were treated overnight with 0.2 µM forskolin and 5 µM IBMX (A) or with 5 µM of the CFTR potentiator VX-770 (B) and stimulated with F/I. (i) time course, (ii) I/Vs, (iii) current density.

(C, D) Cells expressing CFTR only (Ci), CFTR+FRB+PLA1a1-FKBP (Cii) or NBCe1-B+FRB+PLA1a1-FKBP (D) were treated with 0.5 µM rapamycin after current stimulation. The pairs shown in the columns are before (black) and after treatment with rapamycin (red).

(E) Current was initiated by the addition of HCO_3^- to cells expressing vector (empty columns) or E-Syt3 (filled columns) and NBCe1-B alone (black), NBCe1-B+IRBIT (red), NBCe1-B(3R/3A) mutant (brown), NBCe1-B(Δ1-95) (green), NBCe1-A (blue) or NBCe2-C (purple). (i) current density and (ii) example I/Vs.

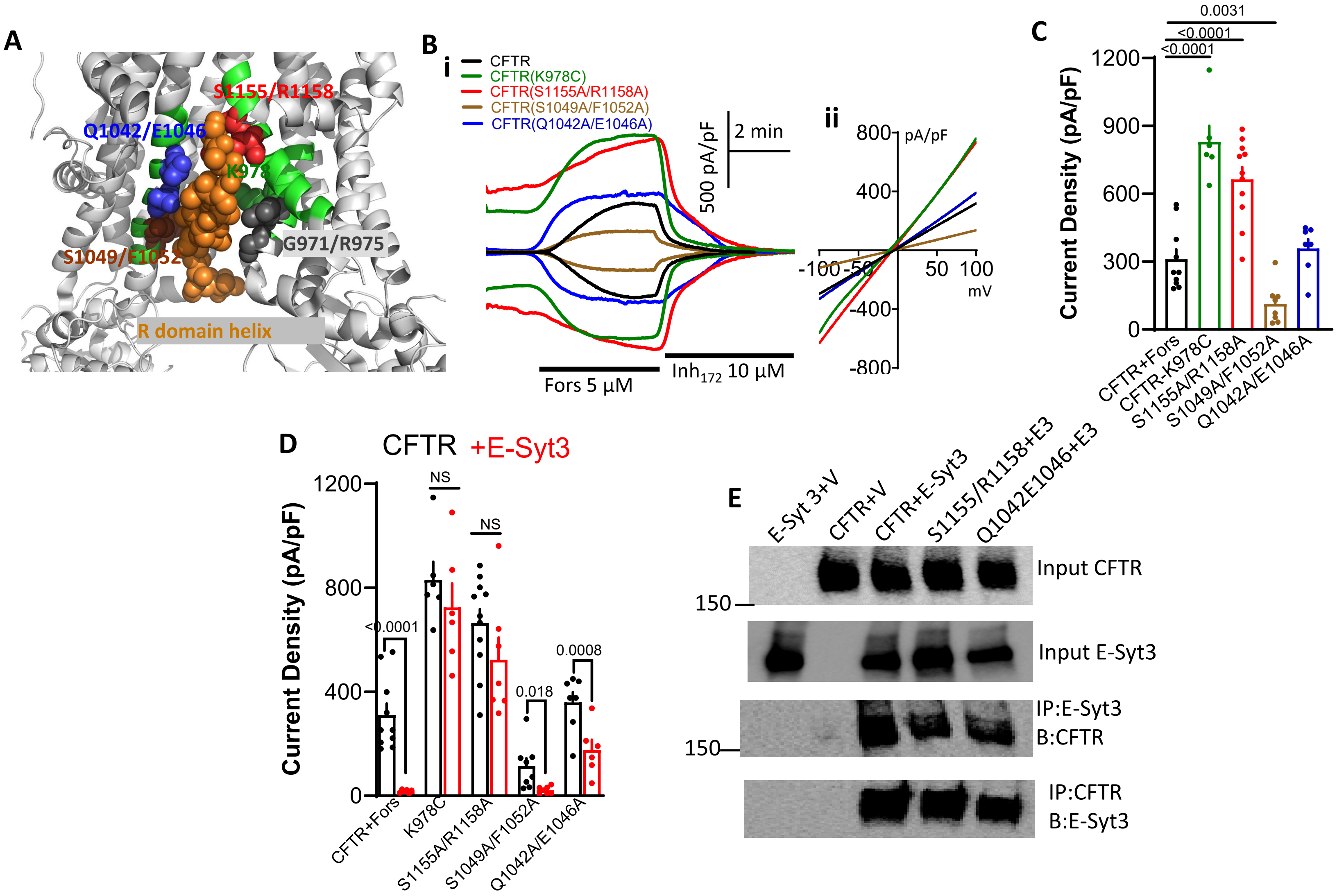

Appendix Figures S14: The R domain as an autoinhibitory domain of CFTR.

(A) The CFTR residues that contact the R domain and mutated in the current studies.

(Bi-ii, C) Example traces (Bi), I/Vs (Bii) and current density (C), for the indicated constitutively active CFTR mutants.

(D) Current density of the CFTR mutants when expressed with vector (black) or with E-Syt3 (red).

(E) The constitutively active CFTR mutants Co-IP with E-Syt3 similar to CFTR.

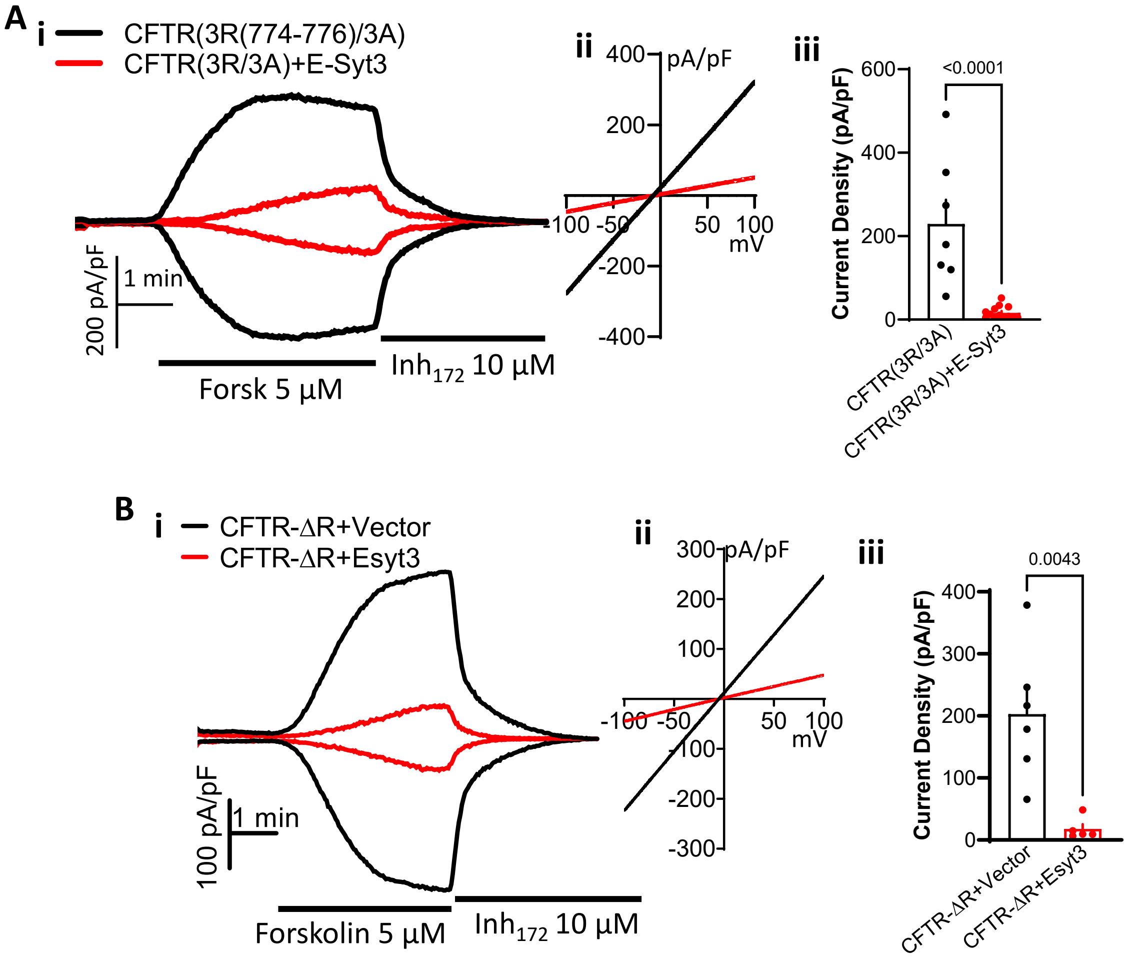

Appendix Figures S15: The R domain does not mediate inhibition of CFTR by E-Syt3.

(A-B) Current was measured with CFTR(R774A/R775A/R776A) (A) and CFTR(ΔRD) (deletion of aa 635-834) (B) when expressed with vector (black) or E-Syt3 (red). (i) current traces, (ii) I/Vs, (iii) current density.

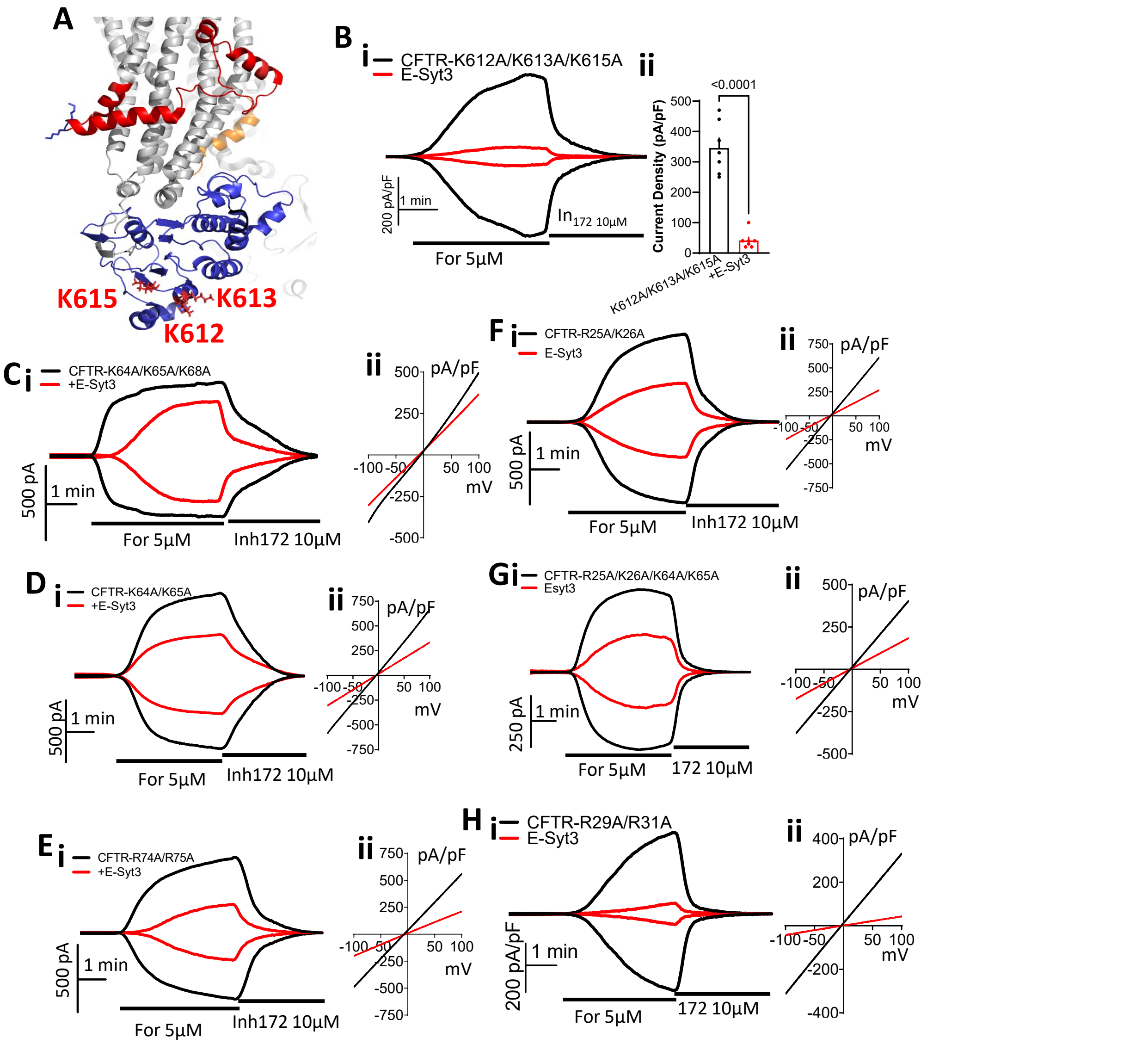

Appendix Figures S16: Effect of CFTR NBD1 mutants, current and i/v for lasso mutants.

(A) The NBD1 surface positively charged residues that tested for inhibition by E-Syt3.
(Bi-ii) Example traces (Bi), and current density (Bii) for CFTR(K612A/K613A/K615A) alone (black) and with E-Syt3 (red).
(C-H) Example current traces (i) and example I/Vs (ii) for the indicated CFTR mutant when expressed with empty vector (black and with E-Syt3 (red).

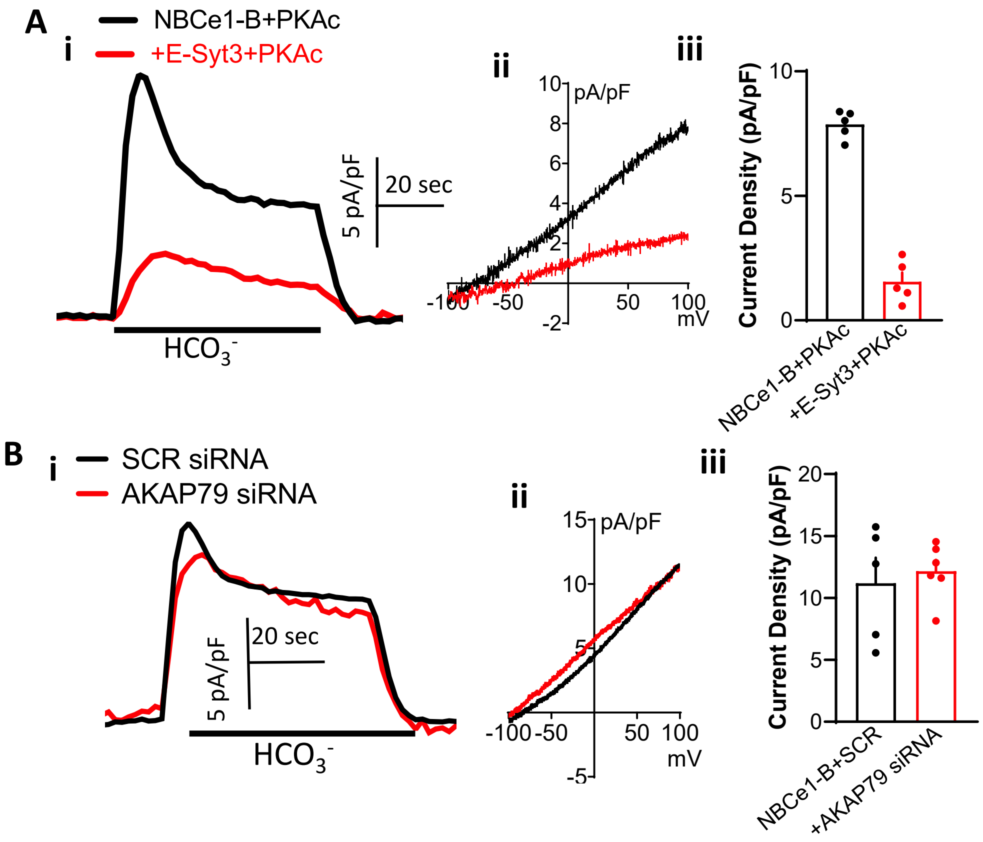

Appendix Figures S17: Lack of a role of PKAc and AKAP79 in NBCe1-B activity.

(Ai-iii, Bi-iii) NBCe1-B current in the presence and absence of E-Syt3 was measured with pipette solution containing the catalytic subunit of PKA (PKAc) (A) and cells treated with scrambled or AKAP79 siRNA (B). (i) current traces, (ii) I/Vs, (iii) current density.

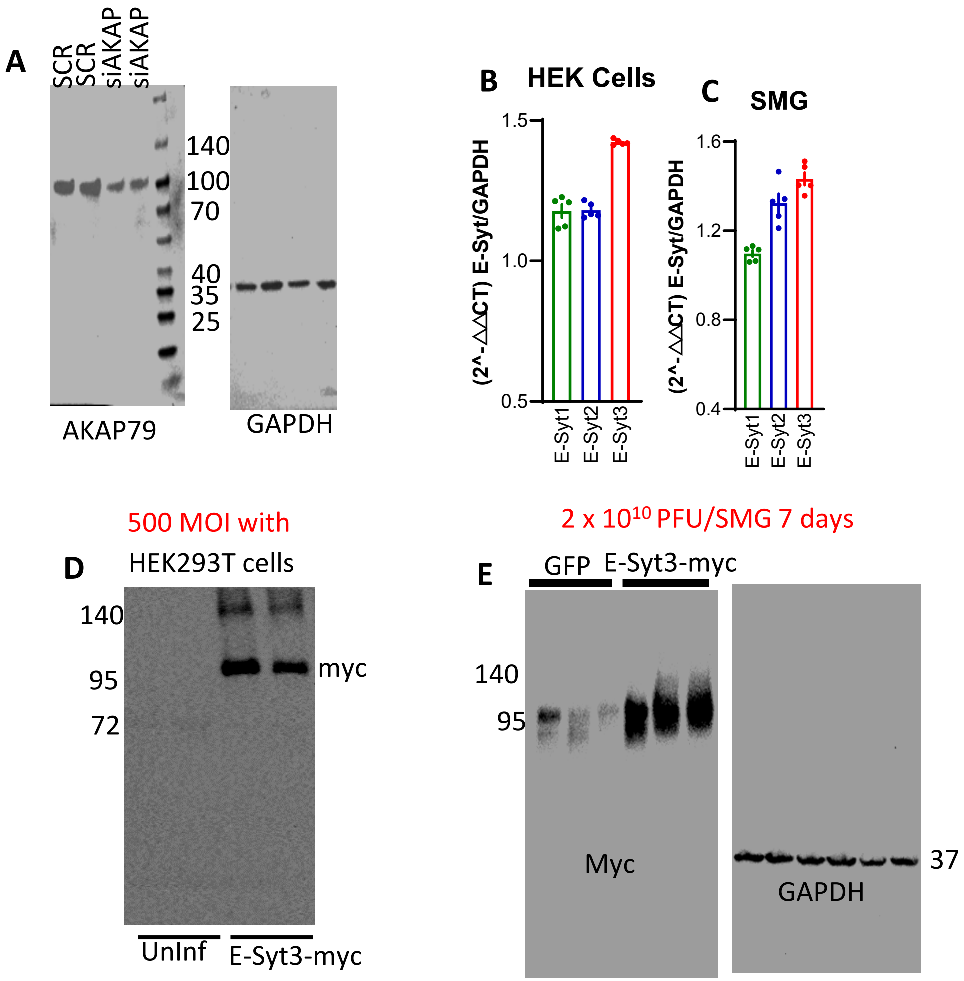

Appendix Figures S18: Effect of siAKAP79, expression of E-Syts mRNA and treatment with viruses carrying E-Syt3 in HEK cells and SMG glands *in vivo*.

(A) Effect of siAKAP79 on the level of native AKAP79 protein. GAPDH is the loading control.
(B, C) the relative level of the E-Syts was determined by RT-PCR of mRNA obtained from HEKT cells (B) or mice SMG (C).
(D) Protein expression of E-Syt3-Myc in HEK293T cells infected with 500 MOI of Adenovirus carrying GFP (control) or E-Syt3-Myc.
(E) Level of E-Syt3-myc in SMG obtained from 3 mice infected with viruses carrying cDNA for expression of GFP (control) or E-Syt3-myc. Left blot probed with anti-myc and right blot probed with DAPDH (loading control).

Appendix Table S1: List constructs and the primers used to generate them.

E-Syt3 (R853/R854/K855A)	AGTAGGCCACTTGGCTCACACGCAGCAGCGGAGTTAGGA AAAGTACTGATT<br>AATCAGTACTTTTCCTAACTCCGCTGCTGCGTGTGAGCCA AGTGGCCTACT
ESyt3 (K123/I124A)	GAGCGGGTCGAGTGGGCCAACGCGGCCATCTCTCAGACC TGGCCCTAC<br>GTAGGGCCAGGTCTGAGAGATGGCCGCGTTGGCCCACTC GACCCGCTC
E-Syt3 (K174S)	TGTCCCAGGGTCAACGGTGTCGCGGCACACACTAATACGT GCAAC<br>GTTGCACGTATTAGTGTGTGCCGCGACACCGTTGACCCTG GGACA
E-Syt3ΔC2C (1-754S)	CTCAGGCGACGGCAGCTGATTGACTTATCAAAAGAAG<br>CTTCTTTTGATAAGTCAATCAGCTGCCGTCGCCTGAG
E3C2C (754-886)	AGAAGCCCGTCGACGGTACCGCTCGAGCGGGATCCACCG GTCATGGTG<br>CACCATGACCGGTGGATCCCGCTCGAGCGGTACCGTCGA CGGGCTTCT
E-Syt3ΔSMP (Δ 114-291)	CGGGGCCAGCACCTGCGCTTCCCT<br>AGGGAAGCGCAGGTGCTGGCCCCG

ESyt3(E2SMP)	ATGTGGCCCTACCTAAGCATGTTCATCGAAAGCAAGTTCC GGGAGACAACTGAGCCCAAGATCCGAGAG<br><br>CTCTCGGATCTTGGGCTCAGTTGTCTCCCGGAACTTGCTT TCGATGAACATGCTTAGGTAGGGCCACAT<br><br>CCCAAGATCCGAGAGGCCAGCATCCACCTGAGGACCTTTA CCTTTACCAAGCTCTACGTTGGACAGAAGTGTCTCAGGGT CAACGGTGTC<br><br>GACACCGTTGACCCTGAGACACTTCTGTCCAACGTAGAGC TTGGTAAAGGTAAAGGTCCTCAGGTGGATGCTGGCCTCTC GGATCTTGGG<br><br>ACTGTGGACCTGCAGATCTGCTTTATCGGGGACTGTGAGA TCAGTCTGGAGCTGCAGAAGATTCAGGCT<br><br>AGCCTGAATCTTCTGCAGCTCCAGACTGATCTCACAGTCC CCGATAAAGCAGATCTGCAGGTCCACAGT<br><br>AACCTGCTGGATGCGCCGGGATTAAATGATTTGTCAGACA GCATAATTGAGGACATCATTGCCACCTACCTGGTGCTGCC CAACCGTGTG<br><br>CACACGGTTGGGCAGCACCAGGTAGGTGGCAATGATGTC CTCAATTATGCTGTCTGACAAATCATTTAATCCCGGCGCAT CCAGCAGGTT<br><br>AACCTGCTGGATGCGCCGGGATTAAATGATTTGTCAGACA GCATAATTGAGGACCTCATTGCCACCCAC<br><br>GTGGGTGGCAATGAGGTCCTCAATTATGCTGTCTGACAAA TCATTTAATCCCGGCGCATCCAGCAGGTT<br><br>TCAGACAGCATAATTGAGGACCTCATTGCCACCTACCTGG TGCTGCCCAACCGTGTG<br><br>CACACGGTTGGGCAGCACCAGGTAGGTGGCAATGAGGTC CTCAATTATGCTGTCTGA

ESyt2(E3SMP)	ATGTGGCCTTTCATTTGCCAAATTATGGAGAAGTTGTTTCG AGAAAAGCTAGAACCAGCCGTGCGGGGAAAAAACACCCA CCTTAGCACC<br><br>GGTGCTAAGGTGGGTGTTTTTTCCCCGCACGGCTGGTTCT AGCTTTTCTCGAAACAACTTCTCCATAATTTGGCAAATGAA AGGCCACAT<br><br>TTTAGTTTCACGAAGGTCGACTTTGGCCAGCAGCCCCCCA GGATCAATGGTGTTAAGGTA<br><br>TACCTTAACACCATTGATCCTGGGGGGCTGCTGGCCAAAG TCGACCTTCGTGAAACTAAA<br><br>ATTATTTTGGACCTTCAGATTAGTTATGTAGGAAATTGTGA GATTGATGTCGAGATCAAACGATATTTTTGT<br><br>ACAAAAATATCGTTTGATCTCGACATCAATCTCACAATTTC CTACATAACTAATCTGAAGGTCCAAAATAAT<br><br>AATCTTCTGGATGTCCCTGGAATTAATGGTGTATCAGATAC TTTGTTGTTGGATATAATATCAAACTAT<br><br>ATAGTTTGATATTATATCCAACAACAAAGTATCTGATACAC CATTAATTCCAGGGACATCCAGAAGATT<br><br>GTATCAGATACTTTGTTGTTGGATCTACTATCAAACCATCT GGTGCTTCCCAATCGAATC<br><br>GATTCGATTGGGAAGCACCAGATGGTTTGATAGTAGATCC AACAACAAAGTATCTGATAC

E1SMP X (1-313)	CGATTACTGGTGCCCCTTTAGCCTGACCTTCAAGATGTG<br><br>CACATCTTGAAGGTCAGGCTAAAGGGGCACCAGTAATCG
E2SMP X (1-370)	TCAGACCCCTATGGAATCTAAAGAGTTGGCAACCAAATC GATTTGGTTGCCAACTCTTTAGATTCCATAGGGGTCTGA
E3SMP X (1-293)	GATCTGACCAACCTGCGCTAACCTCTGCCCTGTGGGGTG CACCCCACAGGGCAGAGGTTAGCGCAGGTTGGTCAGATC
E2C2C (786-921)	GCACTTGCTGGAGACGCGTGCAGAGCAGCTGG CCAGCTGCTCTGCACGCGTCTCCAGCAAGTGC

E3SMP (114-293)	AGCGGACCGACGCGTATGCTTGACAATGAACGCGAGTTC ATC<br>CTCCAGCGGCCGCGTCACCCCACAGGGCAGAGGGAA
NBCe1B His-1-95	TTTGAAGAAAAAGTGGAACAGATCTGGGAAAGATGGAGCA AG<br>CTTGCTCCATCTTTCCCAGATCTGTTCCACTTTTTCTTCAAA
CFTR (R25/K26A)	AGCTGGACCAGACCAATTTTGGCGGCAGGATACAGACAG<br>CGCCTGGAA<br>TTCCAGGCGCTGTCTGTATCCTGCCGCCAAAATTGGTCTG GTCCAGCT
CFTR (K64A/K65A)	TGGGATAGAGAGCTGGCTTCAGCGGCAAATCCTAAACTCA TTAATGCC<br>GGCATTAATGAGTTTAGGATTTGCCGCTGAAGCCAGCTCT<br>CTATCCCA
CFTR (R74A/R75A)	CCTAAACTCATTAATGCCCTTGCGGCATGTTTTTTCTGGAG ATTTATG<br>CATAAATCTCCAGAAAAAACATGCCGCAAGGGCATTAATG AGTTTAGG

CFTR(R774A/R775A/R7 76A)	ACTGGCCCCACGCTTCAGGCAGCAGCGGCGCAGTCTGTC CTGAACCTGATGACA<br>TGTCATCAGGTTCAGGACAGACTGCGCCGCTGCTGCCTG AAGCGTGGGGCCAGT
CFTR (Q1042A/E1046A)	CAAACCTCACAGCAACTCAAAGCACTGGAATCTGCAGGCA GGAGTCCAATTTTCACT<br>AGTGAAAATTGGACTCCTGCCTGCAGATTCCAGTGCTTTG AGTTGCTGTGAGGTTTG
CFTR (S1049A/F1052A)	CAACTGGAATCTGAAGGCAGGGCACCAATTGCCACTCATC TTGTTACAAGCTTA<br>TAAGCTTGTAACAAGATGAGTGGCAATTGGTGCCCTGCCT<br>TCAGATTCCAGTTG
CFTR (S1155/R1158A)	AACTCCAGCATAGATGTGGATGCCTTGATGGCATCTGTGA GCCGAGTCTTTAAG<br>CTTAAAGACTCGGCTCACAGATGCCATCAAGGCATCCACA TCTATGCTGGAGTT
CFTR (K612A/K613A/K615 A)	ACTTCTAAAATGGAACATTTAGCGGCAGCTGACGCAATATT AATTTTGCATGAAGGT<br>ACCTTCATGCAAAATTAATATTGCGTCAGCTGCCGCTAAAT GTTCCATTTTAGAAGT
CFTR (R29/R31A)	CCAATTTTGAGGAAAGGATACGCACAGGCCCTGGAATTGT CAGACATATAC<br>GTATATGTCTGACAATTCCAGGGCCTGTGCGTATCCTTTC<br>CTCAAAATTGG

CFTR K52/R55/R59	GCATTGGAAGCAGAATGGGATGCAGAGCTGGCTTCAAAG AAAAATCCT<br><br>AGGATTTTTCTTTGAAGCCAGCTCTGCATCCCATTCTGCTT CCAATGC
CFTR (K64/K65/K68 A)	TGGGATAGAGAGCTGGCTTCAGCGGCAAATCCTGCACTCA TTAATGCC GGCATTAATGAGTGCAGGATTTGCCGCTGAAGCCAGCTCT CTATCCCA
siE-Syt3 mouse	mm.Ri.Esyt3.13.3
siE-Syt3 human	hs.Ri.ESYT3.13.3
E3SMP(K207EI/208E)	GAG ATC AGT GTG GAG CTG CAG GAG GAA CAG GCT GGT GTG AAC GGG ATC CAG<br><br>CTG GAT CCC GTT CAC ACC AGC CTG TTC CTC CTG CAG CTC CAC ACT GAT CTC
